# Effects of Fatty Acid Amide Hydrolase Inhibitors Acute Administration on the Positive and Cognitive Symptoms of Schizophrenia in Mice

**DOI:** 10.1007/s12035-019-1596-0

**Published:** 2019-04-19

**Authors:** Marta Kruk-Slomka, Izabela Banaszkiewicz, Tomasz Slomka, Grazyna Biala

**Affiliations:** 1 Department of Pharmacology and Pharmacodynamics, Medical University of Lublin, Chodzki 4a Street, 20-093 Lublin, Poland; 2 Department of Mathematics and Medical Biostatistics, Medical University of Lublin, Jaczewskiego 4 Street, 20-954 Lublin, Poland

**Keywords:** Schizophrenia-like symptoms, Endocannabinoids, FAAH, MAGL, MK-801, Mice

## Abstract

The connection between the endocannabinoid system (ECS) and schizophrenia is supported by a large body of research. The ECS is composed of two types cannabinoid (CB: CB1 and CB2) receptors and their endogenous ligands, endocannabinoids. The best-known endocannabinoids, anandamide (AEA) and 2-arachidonoylglycerol (2-AG), are intracellularly degraded by fatty acid hydrolase (FAAH) and monoacylglycerol lipase (MAGL), respectively. Thus, the function of ECS might be modulated in a direct way, through CB receptor ligands or indirectly by FAAH and MAGL inhibitors. We evaluated that the direct influence of ECS, using FAAH (URB 597) and MAGL (JZL 184) inhibitors, on the schizophrenia-like effects in mice. The behavioral schizophrenia-like symptoms were obtained in animals by using N-methyl D-aspartate (NMDA) receptor antagonists, MK-801. An acute administration of MK-801 (0.3 and 0.6 mg/kg) induced psychotic symptoms in rodents, manifested as the increase in locomotor activity, measured in actimeters, as well as the memory impairment, assessed in the passive avoidance (PA) task. We revealed that an acute administration of URB 597, at the dose of 0.3 mg/kg, attenuated MK-801 (0.6 mg/kg)-induced memory impairment. In turn, an acute administration of URB 597 at a higher dose (1 mg/kg) potentiated MK-801 (0.3 mg/kg)-induced memory impairment. Similarly, an acute administration of JZL 184 (20 and 40 mg/kg) intensified an amnestic effect of MK-801 (0.3 mg/kg). Moreover, an acute injection of JZL 184 (1 mg/kg) potentiated hyperlocomotion is provoked by MK-801 (0.3 and 0.6 mg/kg) administration. The present findings clearly indicate that ECS, through an indirect manner, modulates a variety of schizophrenia-like responses in mice.

## Introduction

Schizophrenia is a chronic psychiatric disorder with heterogeneous background and is expressed as a combination of diverse symptoms. The signs of schizophrenia are formally divided into three distinct symptom clusters: positive, negative, and cognitive [1]. Positive symptoms (psychotic ones) refer to hallucinations, delusions, and disorganization. The negative symptoms are characterized by social withdrawal and anhedonia. The cognitive symptoms include deficits in semantic and explicit memory and deficits in attention and working memory [2, 3].

Schizophrenia is a widely prevalent psychiatric disorder whose etiology and management has been still in large part unknown. Pharmacological studies of antipsychotic drugs have fueled hypothesis focused on neurotransmitter mechanisms which underlying pathophysiology of schizophrenia. They include alterations in dopamine (DA), glutamate (Glu), acetylcholine (ACh), serotonin (5-HT), and gamma-aminobutyric acid (GABA) neurotransmission [4, 5].

The most known hypotheses of schizophrenia are based on glutamatergic neurotransmission dysfunction. Numerous evidence from clinical pharmacology, physiology, and brain imaging have recommended that altered glutamatergic functions might lead to clinical features typical for schizophrenia, especially positive and cognitive symptoms [6]. Glu is present throughout the entire nervous system and is the main excitatory neurotransmitter in mammals [5]. The potential relevance of Glu in the pathophysiology of schizophrenia was discovered by research with N-methyl D-aspartate (NMDA) receptors antagonists such as phencyclidine (PCP), ketamine, or dizocilpine (MK-801). These compounds administered to healthy subjects can induce psychotic symptoms and cognitive deficits which mimic those observed in schizophrenia [7]. Many evidences suggest that schizophrenia involves a diminished function or density of NMDA receptors caused by abnormalities in Glu neurotransmission [5]. In postmortem studies, a decrease in NMDA receptors density in the prefrontal cortex and hippocampus has been observed [8]. Moreover, a reduction in the density of dendritic spines, forming excitatory glutamatergic synapses which also may affect inadequate glutamate neurotransmission has been shown [9]. Given this, NMDA receptor antagonists have been extensively used to model aspects of the disease in laboratory animals and have provided a useful preclinical tool for testing novel treatment strategies with strong predictive validity and a growing construct validity potential [10, 11].

Several lines of evidence point to a close relationship between the endocannabinoid system (ECS) and schizophrenia, as cannabis use may precipitate or exacerbate the symptoms of this disease. Following that, over the recent years, significant advances have been made in the ECS as a new target for therapy of schizophrenia. The ECS represents one of the most significant neurotransmitter systems in the brain and plays a relevant role in many physiological processes. The ECS is comprised of cannabinoid receptors (CB1 and CB2), endogenous cannabinoids (endocannabinoids), and the enzymes responsible for the synthesis and degradation of the endocannabinoids. The best-known endocannabinoids are anandamide (AEA) and 2-arachidonoylglycerol (2-AG). Endocannabinoids, including AEA and 2-AG, activate both G-protein coupled CB receptors, but also they can target other non-CB1/CB2 receptors, showing a complex pharmacological profile [12, 13]. The biological actions of the AEA and 2-AG are terminated by enzymatic hydrolysis of these lipids via FAAH and MAGL, respectively [14]. So far, there are two main approaches to the modulation of endocannabinoid functioning. The function of ECS might be modulated in a direct way, by ligands of CB (CB1 or/and CB2) receptors, or indirectly by FAAH or MAGL inhibitors [15]. Following that, this plasticity of ECS signaling opened the way to the development of drugs that counteract the action of endocannabinoids, by inhibiting their inactivation or their binding to the receptor, respectively. However, their effectiveness remains still controversial.

Despite a growing consensus that the cannabinoids can modulate schizophrenia-like symptoms [16, 17], as well as that there are some findings of indirect modulation in endocannabinoids levels gain with FAAH or MAGL inhibitors on memory or locomotion in rodents [18–22], there is a distinct lack of evidence regarding to the influence of these inhibitors on the specific schizophrenia-like responses in animals. Therefore, using an animal model of schizophrenia (based on glutamate hypothesis of schizophrenia), in the current study, we sought to investigate this issue by examining how indirect alteration of endocannabinoids level affect the behavioral responses connected with hypofunction of glutamate neurotransmission. We used chemical compounds which are selective enzymes’ inhibitors, such as URB 597 which is specific for FAAH, and JZL 284, an inhibitor for MAGL. To trigger a Glu dysfunction, we employed MK-801, a NMDA receptor antagonist, which is a commonly accepted model of schizophrenia, and provokes a wide range of schizophrenia-like symptoms in rodents (e.g., learning and memory deficits or hyperlocomotion).

The findings of these experiments will enlarge the knowledge concerning the indirect involvement of ECS in the schizophrenia-like responses in mice, including cognitive disorders and hyperlocomotion.

## Materials and Methods

### Animals

The experiments were carried out on naive male Swiss mice (Farm of Laboratory Animals, Warszawa, Poland) weighing 20–30 g. The animals were maintained under standard laboratory conditions (12-h light/dark cycle, room temperature at 21± 1 °C) with free access to tap water and laboratory feeding (Agropol, Motycz, Poland) in their home cages, and adapted to the laboratory conditions for at least 1 week. Each experimental group consisted of 8–12 animals. All behavioral experiments were performed between 8:00 and 15:00, and were conducted according to the National Institute of Health Guidelines for the Care and Use of Laboratory Animals and to the European Community Council Directive for the Care and Use of laboratory animals of 22 September 2010 (2010/63/EU), and approved by the local ethics committee.

### Drugs

The compounds which were tested:

URB 597 (0.1, 0.3, 1 mg/kg) (Tocris, USA)—FAAH inhibitor

JZL 184 (1, 4, 8, 20, 40 mg/kg) (Tocris, USA)—MAGL inhibitor

MK-801 (0.3, 0.6 mg/kg) (Tocris, USA)—NMDA receptor antagonist

All compounds were suspended in a 1% solution of Tween 80 (Sigma, St. Louis, MO, USA) in saline solution (0.9% NaCl) and administered intraperitoneally (ip) at a volume of 10 ml/kg. Fresh drug solutions were prepared on each day of experimentation. Control groups received injections of saline with Tween 80 (vehicle) at the same volume and by the same route of administration.

Experimental doses of drugs used and procedures were selected on the basis of literature data [23–28] and our previous experiments [16, 17, 29].

### Experimental Procedures

We used a pharmacological animal model of schizophrenia, i.e., an administration of a NMDA receptor antagonist, MK-801. The used procedure is commonly accepted [23, 27] and confirmed in our previous experiments [16, 17, 29]. The experimental procedure is based on the amnestic and psychotic properties of MK-801. An acute administration of MK-801 induced in mice schizophrenia-like symptoms, manifested as the increase in locomotor activity (correlation with the positive symptoms of schizophrenia in humans), and cognitive disturbances (correlation with the cognitive symptoms of schizophrenia in humans).

In our previously published experiments, we confirmed that an acute injection of MK-801 at the doses of 0.3 and 0.6 mg/kg diminished the short term as well as long-term acquisition, consolidation/retention, and/or retrieval of memory and learning in the PA task [29]. In other experiments, we also confirmed that an acute administration of MK-801 at the doses of 0.3 and 0.6 mg/kg significantly increased the locomotor activity of mice [16, 17]. Therefore, based on the results obtained from our cited experiments, these two doses of MK-801 (0.3 and 0.6 mg/kg) were then chosen for the provoked cognitive and positive symptoms typical for schizophrenia in mice.

In the presented experiments, we evaluated for the first time the influence of an acute administration of URB 597 and JZL 184 on the above described schizophrenia-like amnestic and psychotic effects in mice, provoked by MK-801. Memory-related responses in mice were measured in the PA task; locomotor activity was measured in actimeters.

#### Memory-Related Responses

The apparatus of the PA consisted of a two-compartment acrylic box with a lighted compartment (10 × 13 × 15 cm) and darkened compartment (25 × 20 × 15 cm). The light chamber was illuminated by a fluorescent light (8 W) and was connected to the dark chamber which was equipped with an electric grid floor. The entrance of animals to the dark box was punished by an electric foot shock (0.2 mA for 2 s).

On the first day of training (pre-test), mice were placed individually into the light compartment and allowed to explore the light box. After 30 s, the guillotine door was raised to allow the mice to enter the dark compartment. When the mice entered the dark compartment, the guillotine door was closed and an electric foot shock (0.2 mA) of 2 s duration was delivered immediately to the animal via grid floor. The latency time for entering the dark compartment was recorded (TL1). The mouse which did not enter spontaneously into the dark box within 300 s was excluded from further tests. Twenty-four hours later, in the subsequent trial (retention), the same mice were again placed individually in the light compartment of the PA apparatus. After a 30-s adaptation period in the light (safe) chamber, the door between the compartments was raised and the time taken to re-enter the dark compartment was recorded (TL2). No foot shock was applied in this trial. Basically, in this kind of procedure, when the mouse did not enter spontaneously into the dark box within 300 s, the test was stopped [25, 29].

#### Locomotion

Locomotion of mice was recorded individually in round actimeter cages (Multiserv, Lublin, Poland; 32 cm in diameter, two light beams) kept in a sound-attenuated experimental room. Two photocell beams, located across the axis, automatically measured animal’s movements. The horizontal locomotor activity, i.e., the number of photocell beam breaks, was automatically measured with a 20-min interval for 200 min [28, 30].

### Treatment

#### For Memory-Related Responses

First, we estimated the influence of URB 597 (0.1, 0.3, and 1 mg/kg) and JZL 184 (4, 8, 20, 40 mg/kg) on the acquisition of long-term memory in mice using the PA test. All tested compounds or vehicle, for the control group, were administered 30 min before the first trial and mice were re-tested after 24 h (Table 1)**.**

**Table 1:** The scheme of fatty acid amide hydrolase inhibitors (JZL 184 and URB 597) or vehicle administration during the assessment of long-term memory acquisition in the PA test in mice

Acquisition of memory					
PA test	Drug administration	Interval	TL1	Interval	TL2
Long-term memory	URB 597 (0.1, 0.3, and 1 mg/kg) or vehicle	30 min	+	24 h	+
	JZL 184 (4, 8, 20, and 40 mg/kg) or vehicle	30 min	+	24 h	+

Next, based on this pilot experiment, we have chosen the non-effective doses of URB 597 and JZL 184 for the next experiment with MK-801.

We evaluated the influence of these compounds on the memory-related disorders induced by MK-801 (0.3 and 0.6 mg/kg) in the PA task. Non-effective doses of URB 597 (0.3 and 1 mg/kg), JZL 184 (20 and 40 mg/kg), or vehicle were administered acutely 15 min before an acute injection of MK-801 (0.3 and 0.6 mg/kg) or vehicle. Fifteen minutes after the last injection, the mice were tested in PA during the first trial and re-tested 24 h later, for the assessment of long-term memory acquisition (Table 2).

**Table 2:** The scheme of fatty acid amide hydrolase inhibitors (JZL 184 and URB 597) and MK-801 co-administration during the assessment of long-term memory acquisition in the PA test in mice

Acquisition of memory							
PA test	Drug administration	Interval	Drug administration	Interval	TL1	Interval	TL2
Long-term memory	URB 595 (0.3 or 1 mg/kg) or vehicle	15 min	MK-801 (0.3 or 0.6 mg/kg) or vehicle	15 min	+	24 h	+
	JZL 184 (20 or 40 mg/kg) or vehicle	15 min	MK-801 (0.3 or 0.6 mg/kg) or vehicle	15 min	+	24 h	+

#### For Psychotic-Like Symptoms

Similarly, as in the case of memory-related effects, first we estimated the influence of an acute administration of URB 597 (0.1, 0.3, and 1 mg/kg), JZL 184 (1, 4, 8, 20, 40 mg/kg), or vehicle for the control group on the locomotion of mice in the actimeters. Horizontal locomotor activity was measured immediately after injection of tested compounds (Table 3).

**Table 3:** The scheme of fatty acid amide hydrolase inhibitors (JZL 184 and URB 597) or vehicle administration during the assessment of locomotor activity of mice

Locomotor activity		
Actimeters		
Drug administration	Interval	The number of photocell beam breaks (0–200 min)
URB 597 (0.1, 0.3, and 1 mg/kg) or vehicle	Immediately	+
JZL 184 (1, 4, 8, 20, and 40 mg/kg) or vehicle	Immediately	+

In the next stage, we assessed the impact of an acute administration of a non-effective dose of URB 597 (1 mg/kg) or JZL 184 (1 mg/kg) on the hyperlocomotion of mice provoked by an acute MK-801 (0.3 and 0.6 mg/kg). For this purpose, URB 597, JZL 184, or vehicle was administered 15 min before the injection of MK-801 or vehicle. The mice were then tested in actimeters immediately after the last injection (Table 4).

**Table 4:** The scheme of fatty acid amide hydrolase inhibitors (JZL 184 and URB 597) and MK-801 co-administration during the assessment of locomotor activity of mice

Locomotor activity				
Actimeters				
Drug administration	Interval	Drug administration	Interval	The number of photocell beam breaks (0–200 min)
URB 595 (0.3 or 1 mg/kg) or vehicle	15 min	MK-801 (0.3 or 0.6 mg/kg) or vehicle	Immediately	+
JZL 184 (20 or 40 mg/kg) or vehicle	15 min	MK-801 (0.3 or 0.6 mg/kg) or vehicle	Immediately	+

In the presented experiments, we used independent groups of mice for each kind of behavioral experiments (a separate group of mice for the assessment of memory-related effects and a separate group of mice for the assessment of locomotor activity) for each drug and dose.

### Statistical Analysis

The statistical analysis were performed using one-way analysis of variance (ANOVA) or two-way ANOVA—for the factors of pretreatment (URB 597 or JZL 184), treatment (MK-801), and pretreatment/treatment interactions for the memory-related responses or for the factors of time, drugs, and time/drugs interactions for the psychotic-like symptoms.

*Post hoc* comparison of means was carried out with the Tukey’s test (for one-way and two-way ANOVA) for multiple comparisons, when appropriate. The data were considered statistically significant at a confidence limit of *p* < 0.05. ANOVA analysis with Tukey’s test was performed using the GraphPad Prism version 5.00 for Windows, GraphPad Software, San Diego California USA, www.graphpad.com.

For the memory-related responses, the changes in PA performance were expressed as the difference between retention and training latencies and were taken as a latency index (LI). LI was calculated for each animal and reports as the ratio:

LI = TL2-TL1/TL1

TL1 – The time taken to enter the dark compartment during the training

TL2 – The time taken to re-enter the dark compartment during the retention [31]

For the psychotic-like symptoms, the horizontal locomotor activity, i.e., the number of photocell beam breaks, was measured.

## Results

### Memory-Related Responses

First, we evaluated the influence of an acute administration of FAAH and MAGL inhibitors on the long-term memory acquisition, and then we assessed the impact of these inhibitors on the memory impairment provoked by an acute injection of MK-801.

#### The Influence of an Acute Injection of URB 597 on the Acquisition of Long-term Memory in Mice in the PA Test

**Fig. 1:**
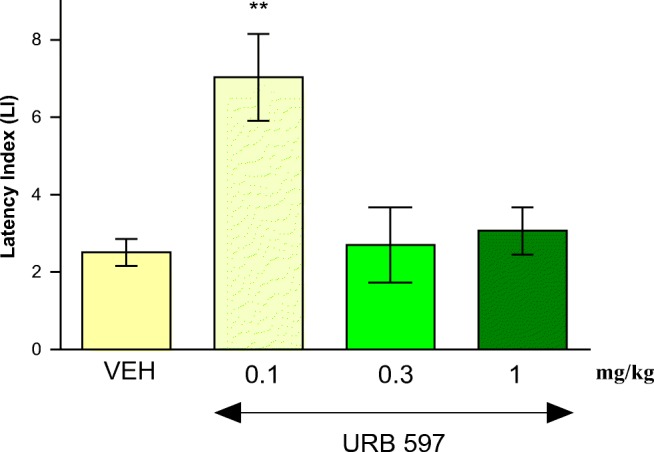
Effects of an acute URB 597 (URB) or vehicle (VEH) administration on the latency index (LI) during the long-term acquisition trial using the PA test in mice. URB (0.1, 0.3, 1 mg/kg; ip) or VEH, for the control group, was injected 30 min before the first trial and mice were then re-tested 24 h later. *n* = 8–9; the means ± SEM; **p < 0.01 for URB (0.1) vs. VEH; Tukey’s test

One-way ANOVA revealed that administration of acute ip doses of URB 597 (0.1, 0.3, and 1 mg/kg) had a statistically significant effect on LI values for long-term memory acquisition [*F*(3,33) = 6.508; *p* = 0.0016]. Indeed, the *post hoc* Tukey’s test confirmed that the treatment with URB 597 (0.1 mg/kg) significantly increased LI values in mice compared to those in the vehicle-treated control group (*p* < 0.01) (Fig. 1), indicating that URB 597, at this used dose, improved long-term acquisition of memory and learning processes in PA test in mice.

#### The Influence of an Acute Injection of JZL 184 on the Acquisition of Long-term Memory in Mice in the PA Test

**Fig. 2:**
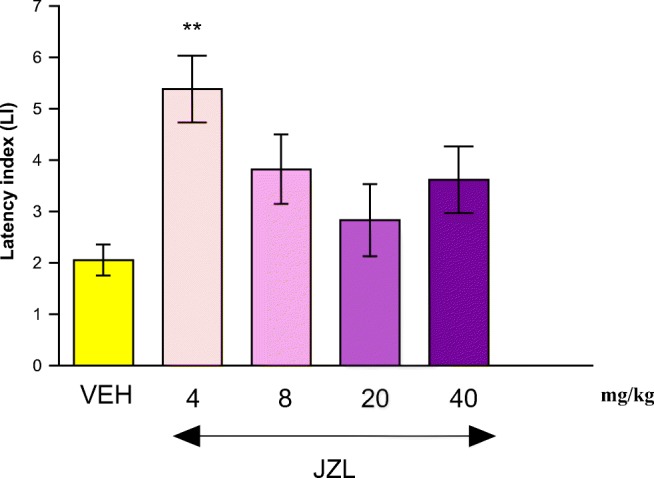
Effects of an acute JZL-184 (JZL) or vehicle (VEH) administration on the latency index (LI) during the long-term acquisition trial using the PA test in mice. JZL (4, 8, 20, and 40 mg/kg; ip) or vehicle (VEH), for the control group, was injected 30 min before the first trial and mice were then re-tested 24 h later. *n* = 8; the means ± SEM; **p < 0.01 for JZL (4) vs. VEH; Tukey’s test

One-way ANOVA revealed that administration of acute ip doses of JZL 184 (4, 8, 20, 40 mg/kg) had a statistically significant effect on LI values for long-term memory acquisition [*F*(4,40) = 4.251; *p* = 0.0064]. Indeed, the *post hoc* Tukey’s test confirmed that the treatment with JZL 184 (4 mg/kg) significantly increased LI values in mice compared to those in the vehicle-treated control group (*p* < 0.01) (Fig. 2), indicating that JZL 184, at this used dose, improved long-term acquisition of memory and learning processes in PA test in mice.

Based on the results obtained from these pilot experiments, the non-effective doses of URB 597 (0.3 and 1 mg/kg) and JZL 184 (20 and 40 mg/kg) were then chosen for the next behavioral experiments evaluating the influence of these FAAH and MAGL inhibitors on the memory impairment, provoked by an acute injection of MK-801 (0.3 or 0.6 mg/kg), using the PA test in mice.

#### The Influence of the Administration of URB 597 on the Memory Impairment Provoked by an Acute Administration of MK-801 in the PA Test in Mice

**Fig. 3:**
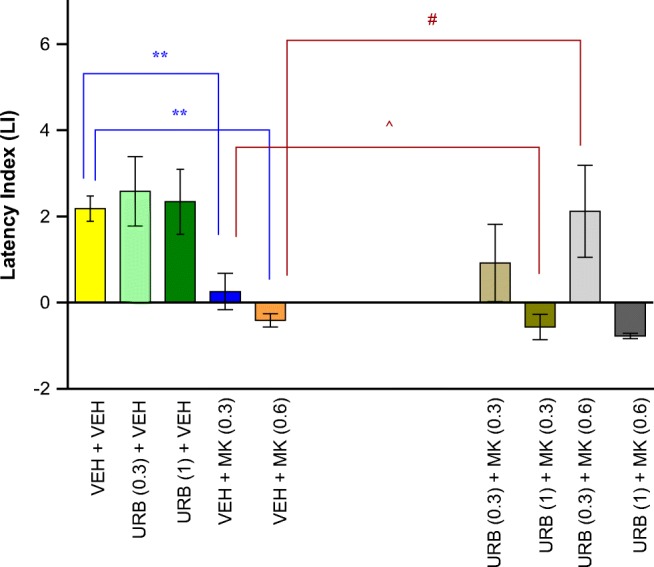
Influence of an acute administration of URB 597 (URB) on the memory impairment induced by MK-801 (MK), expressed as latency index (LI) during the long-term acquisition using the PA test in mice. Non-effective doses of URB (0.3 or 1 mg/kg) or vehicle were administered 15 min prior to vehicle (VEH) or effective doses of MK (0.3 or 0.6 mg/kg). Fifteen minutes after the last injection, the first trial was conducted and 24 h later after the second trial; *n* = 8–11; the means ± SEM; **p < 0.01 for VEH + MK (0.3) vs. VEH + VEH; **p < 0.01 for VEH + MK (0.6) vs. VEH + VEH; ^*p* < 0.05 for URB (1) + MK (0.3) vs. VEH + MK (0.3); #*p* < 0.05 for URB (0.3) + MK (0.6) vs. VEH + MK (0.6); Tukey’s test

For long-term memory acquisition, two-way ANOVA analyses revealed that there was statistically significant effect caused by URB 597 (0.3 or 1 mg/kg) pretreatment [*F*(2,70) = 5.200; *p* = 0.0078] as well as by MK-801 (0.3 or 0.6 mg/kg) treatment [*F*(2,70) = 11.81; *p* < 0.0001], but there was no statistically significant effect caused by interactions [*F*(4,70) = 1.321; *p* = 0.2708]. The *post hoc* Tukey’s test confirmed that MK-801 at the dose of 0.3 and 0.6 mg/kg significantly decreased LI values in mice in the PA test in comparison to the vehicle/vehicle-treated mice, pointing to the amnestic effect of this drug (*p* < 0.01). Moreover, an acute injection of URB 597 (0.3 mg/kg) attenuated the amnestic effect of MK-801 (0.6 mg/kg) (*p* < 0.05); in turn, an acute injection of URB 597 in higher dose (1 mg/kg) potentiated the amnestic effect of MK-801 (0.3 mg/kg) (*p* < 0.05; Tukey’s test) (Fig. 3).

#### The Influence of the Administration of JZL 184 on the Memory Impairment Provoked by an Acute Administration of MK-801 in the PA Test in Mice

**Fig. 4:**
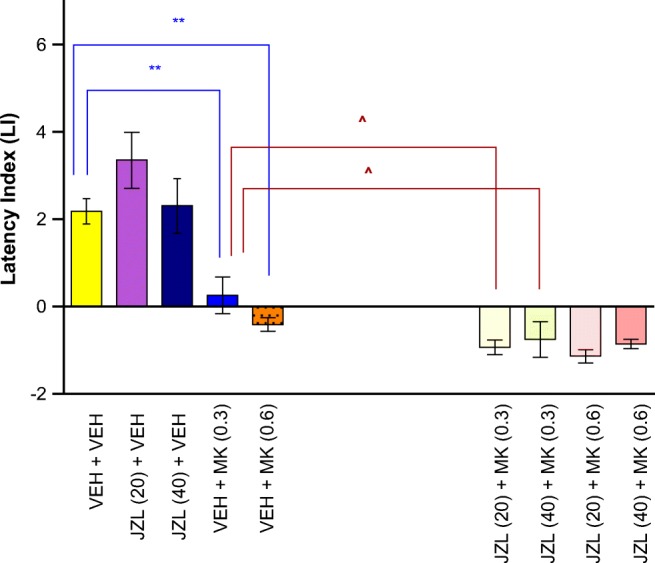
Influence of an acute administration of JZL 184 (JZL) on the memory impairment induced by MK-801 (MK), expressed as latency index (LI) during the long-term acquisition using the PA test in mice. Non-effective doses of JZL (20 or 40 mg/kg) or vehicle were administered 15 min prior to vehicle (VEH) or effective doses of MK (0.3 or 0.6 mg/kg). Fifteen minutes after the last injection, the first trial was conducted and 24 h later after the second trial; *n* = 8–11; the means ± SEM; **p < 0.01 for VEH + MK (0.3) vs. VEH + VEH; **p < 0.01 for VEH + MK (0.6) vs. VEH + VEH; ^*p* < 0.05 for JZL (20) + MK (0.3) vs. VEH + MK (0.3); and ^*p* < 0.05 for JZL (40) + MK (0.3) vs. VEH + MK (0.3); Tukey’s test

For long-term memory acquisition, two-way ANOVA analyses revealed that there was no statistically significant effect caused by JZL 184 (20 or 40 mg/kg) pretreatment [*F*(2,75) = 1.025; *p* = 0.3637], but there was a statistically significant effect caused by MK-801 (0.3 or 0.6 mg/kg) treatment [*F*(2,75) = 73.79; *p* < 0.0001], as well as by interactions [*F*(4,75) = 2.811; *p* = 0.0313]. The *post hoc* Tukey’s test confirmed that MK-801 at the dose of 0.3 and 0.6 mg/kg significantly decreased LI values in mice in the PA test in comparison to the vehicle/vehicle-treated mice, pointing to the amnestic effect of this drug (*p* < 0.01). In turn, an acute injection of JZL 184 (20 and 40 mg/kg) potentiated the amnestic effect of MK-801 (0.3 mg/kg) (*p* < 0.05; Tukey’s test) (Fig. 4).

### Locomotor Activity

First, we evaluated the influence of an acute administration of FAAH and MAGL inhibitors on the locomotion of mice.

#### The Influence of an Acute Injection of URB 597 on the Locomotor Activity in Mice

Two-way ANOVA analyses revealed that there was statistically significant effect caused by time [*F*(10,231) = 23.51; *p* < 0.0001] and URB 597 (0.1, 0.3, and 1 mg/kg) treatment [*F*(3,231) = 13.94; *p* < 0.0001], but there was no statistically significant effect caused by interactions between time and URB 597 treatment [*F*(30,231) = 0.7358; *p* = 0.8415].

The Tukey’s test revealed that an acute injection of URB 597 at the dose of 0.1 mg/kg significantly decreased locomotion in mice between 140 and 200 min of experiments in comparison to the vehicle-treated control group (for 140 and 160 min of experiments *p* < 0.05; for 160–200 min; *p* < 0.01).

Similarly, the Tukey’s test revealed that an acute injection of URB 597 at the dose of 0.3 mg/kg significantly decreased the locomotor activity of mice between 180 and 200 min of the experiment as compared with the vehicle-treated control group (*p* < 0.05).

**Fig. 5:**
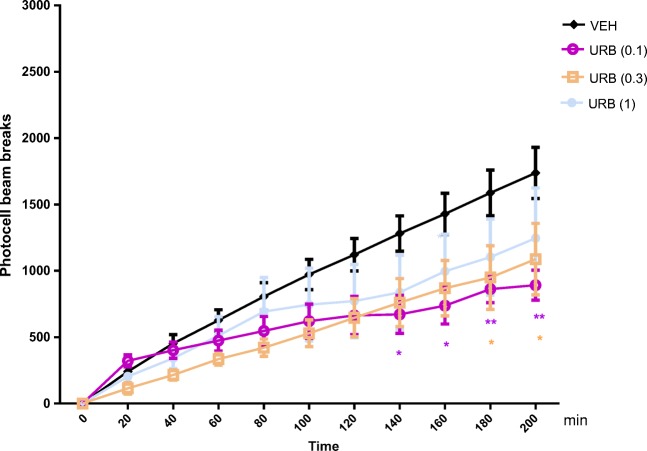
Effects of an acute URB-597 (URB) or vehicle (VEH) administration on the locomotor activity in mice. URB (0.1; 0.3 and 1 mg/kg; ip) or VEH, for the control group, was injected 30 min before the test; *n* = 6; the means ± SEM; *p < 0.05 for URB (0.3) vs. VEH; *p < 0.05 and **p < 0.01 for URB (0.1) vs. VEH; Tukey’s test

URB 597 at the dose of 1 mg/kg had no influence on the locomotor activity of mice in comparison to the vehicle-treated control group (Fig. 5).

#### The Influence of an Acute Injection of JZL 184 on the Locomotor Activity in Mice

Two-way ANOVA analyses revealed that there was statistically significant effect of time [*F*(10,330) = 22.96; *p* < 0.0001] and JZL 184 (1, 4, 8, 20, 40 mg/kg) treatment [*F*(5,330) = 51.47; *p* < 0.0001], as well as of interactions between time and JZL 184 treatment [*F*(50,330) = 2.272; *p* < 0.0001]. The Tukey’s test confirmed that an acute injection of JZL 184 at the range of doses used (4–40 mg/kg) significantly decreased locomotion in mice between 80 and 200 min of experiments in comparison to the vehicle-treated control group:

- For dose of 4 mg/kg*:* 140 min of experiments (*p* < 0.05); 160–200 min of experiments *p* < 0.001).
- For dose of 8 mg/kg*:* 140 min of experiments (*p* < 0.05); 120 min of experiments (*p* < 0.01); 140–200 min of experiments (*p* < 0.001)
- For dose of 20 mg/kg*:* 80 min of experiments (*p* < 0.05); 100 min of experiments (*p* < 0.01); 120–200 min of experiments (*p* < 0.001)
- For dose of 40 mg/kg*:* 80 min of experiments (*p* < 0.05); 100 min of experiments (*p* < 0.01); 120–200 min of experiments (*p* < 0.001)

**Fig. 6:**
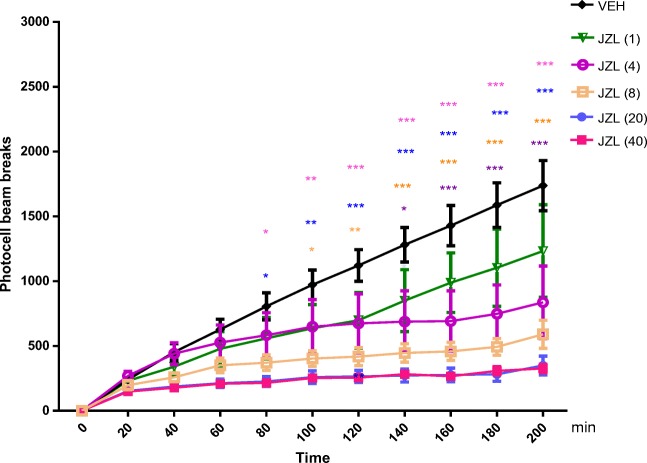
Effects of an acute JZL 184 (JZL) or vehicle (VEH) administration on the locomotor activity in mice. JZL (1, 4, 8, 20, 40 mg/kg; ip) or VEH, for the control group, was injected 30 min before the test; *n* = 6; the means ± SEM; *p < 0.05 and ***p < 0.001 for JZL (4) vs. VEH; *p < 0.05; **p < 0.01 and ***p < 0.001 for JZL (8) vs. VEH; *p < 0.05; **p < 0.01 and ***p < 0.001 for JZL (20) vs. VEH; *p < 0.05; **p < 0.01 and ***p < 0.001 for JZL (40) vs. VEH; Tukey’s test

JZL 184 at the dose of 1 mg/kg had no influence on the locomotor activity of mice in comparison to the vehicle-treated control group (Fig. 6).

In the next stage of experiments, we assessed the impact of tested inhibitors on the hyperlocomotion induced by MK-801. We used a combination of non-effective doses of URB 597 (1 mg/kg) or JZL 184 (1 mg/kg) with an effective dose of MK-801 (0.3 or 0.6 mg/kg).

#### The Influence of an Acute Administration of URB 597 on the Hyperactivity of Mice Provoked by an Acute Administration of MK-801

**Fig. 7:**
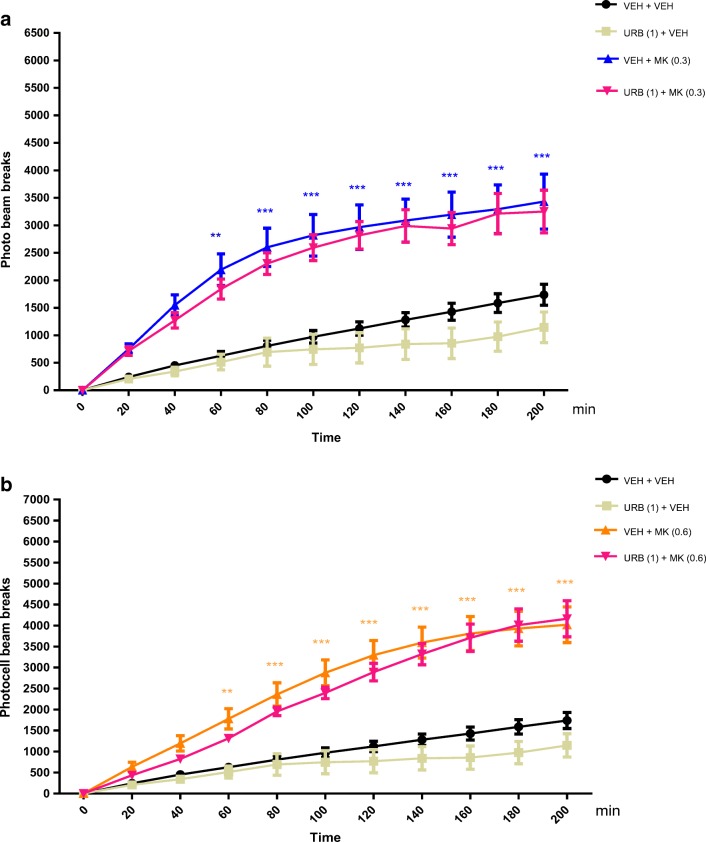
Effects of an acute administration of non-effective dose of URB-597 (URB) on the hyperlocomotion induced by MK-801 (MK) administration. URB (1 mg/kg; ip) or vehicle (VEH) was injected 15 min before MK (0.3 mg/kg; ip) (**a**) or MK (0.6 mg/kg; ip) (**b**); *n* = 6–7; the means ± SEM; **p < 0.01; ***p < 0.001 for VEH + MK (0.3) vs. VEH + VEH; **p < 0.01; ***p < 0.001 for VEH + MK (0.6) vs. VEH + VEH; Tukey’s test

Two-way ANOVA analyses revealed that there was statistically significant effect caused by time [*F*(10,242) = 37.82; *p* < 0.0001], drugs (MK-801 (0.3 mg/kg), and/or URB 597 (1 mg/kg) treatment [*F*(3,242) = 129.1; *p* < 0.0001], as well as by interactions between time and drugs treatment [*F*(30,242) = 2.602; *p* < 0.0001]. The post hoc Tukey’s test confirmed that an acute injection of MK-801 at the dose of 0.3 mg/kg significantly increased locomotor activity of mice between 60 and 200 min of experiment as compared with the vehicle/vehicle-injected control group (for 60 min of experiments, *p* < 0.01; for 80–200 min of experiments, *p* < 0.001). URB 597 (1 mg/kg) had no influence on MK-801 (0.3 mg/kg)-induced hyperactivity (Fig. 7a).

Similarly, two-way ANOVA analyses revealed that there was statistically significant effect of time [*F*(10,242) = 65.63; *p* < 0.0001], drugs (MK-801 (0.6 mg/kg), and/or URB 597 (1 mg/kg) treatment [*F*(3,242) = 176.5; *p* < 0.0001], as well as of interactions between time and drugs treatment [*F*(30,242) = 6.386; *p* < 0.0001]. The post hoc Tukey’s test confirmed that MK-801 at the dose of 0.6 mg/kg significantly increased locomotor activity of mice in actimeters between 60 and 200 min of experiments (for 60 min of experiments, *p* < 0.05; for 80–200 min, *p* < 0.001), in comparison to the vehicle/vehicle-treated mice. URB 597 (1 mg/kg) had no influence on MK-801 (0.6 mg/kg)-induced hyperactivity (Fig. 7b).

#### The Influence of an Acute Administration of JZL 184 on the Hyperactivity of Mice Provoked by an Acute Administration of MK-801

**Fig. 8:**
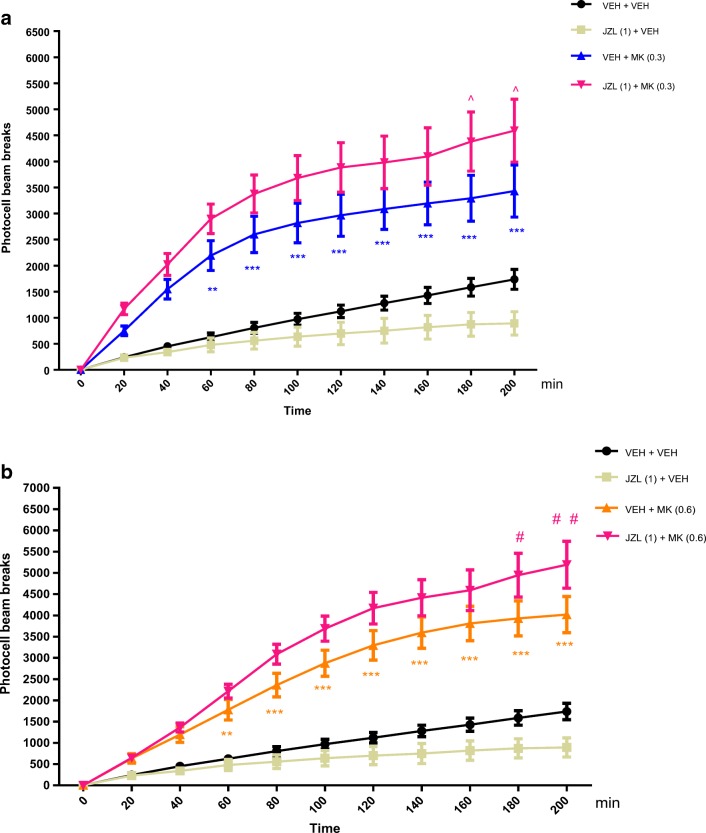
Effects of an acute administration of non-effective dose of JZL 184 (JZL) on the hyperlocomotion induced by MK-801 (MK) administration. JZL (1 mg/kg; ip) or vehicle (VEH) was injected 15 min before MK (0.3 mg/kg; ip) (**a**) or MK (0.6 mg/kg; ip) (**b**); *n* = 6–7; the means ± SEM; **p < 0.01; ***p < 0.001 for VEH + MK (0.3) vs. VEH + VEH; ^*p* < 0.05 for JZL (1) + MK (0.3) vs. VEH + MK (0.3); **p < 0.01; ***p < 0.001 for VEH + MK (0.6) vs. VEH + VEH; #*p* < 0.05; ##*p* < 0.01 for JZL (1) + MK (0.6) vs. VEH + MK (0.6); Tukey’s test

Two-way ANOVA analyses revealed that there was statistically significant effect of time [*F*(10,242) = 34.66; *p* < 0.0001], drugs (MK-801(0.3 mg/kg), and/or JZL 184 (1 mg/kg) treatment [*F*(3,242) = 185.8; *p* < 0.0001], as well as of interactions between time and drugs treatment [*F*(30,242) = 3.553; *p* < 0.0001]. The post hoc Tukey’s test confirmed that an acute injection of MK-801 at the dose of 0.3 mg/kg significantly increased locomotor activity of mice between 60 and 200 min of experiment in comparison to the vehicle/vehicle-treated mice (for 60 min of experiments, *p* < 0.05; for 80–200 min, *p* < 0.001). Moreover, the *post hoc* test confirmed that this hyperactivity provoked by MK-801 (0.3 mg/kg) was attenuated by JZL 184 (1 mg/kg) between 180 and 200 min of experiments (*p* < 0.05) vs. vehicle/MK-801 (0.3 mg/kg)-treated mice) (Fig. 8a).

Similarly, two-way ANOVA analyses revealed that there was statistically significant effect of time [*F*(10,242) = 64.20; *p* < 0.0001], drugs (MK-801(0.6 mg/kg), and/or JZL 184 (1 mg/kg) treatment [*F*(3,242) = 256.8; *p* < 0.0001], as well as of interactions between time and drugs treatment [*F*(30,242) = 8.034; *p* < 0.0001]. The post hoc Tukey’s test confirmed that an acute injection of MK-801 at the dose of 0.6 mg/kg significantly increased locomotor activity of mice between 60 and 200 min of experiment in comparison to the vehicle/vehicle-treated mice (for 60 min of experiments, *p* < 0.05; for 80–200 min, *p* < 0.001). Moreover, the *post hoc* test confirmed that this hyperactivity provoked by MK-801 (0.6 mg/kg) was attenuated by JZL 184 (1 mg/kg) between 180 min (*p* < 0.05) and 200 min (*p* < 0.01) of experiments vs. vehicle/MK-801 (0.6 mg/kg)-treated mice) (Fig. 8b).

## Discussion

The ECS is a key modulator of several physiological functions, including emotional as well as memory and learning processes [32–34]. Several lines of experimental and clinical reports also revealed a clear relationship between CB receptor ligands and schizophrenia-like responses [16, 17, 35]. For example, CB1 receptor agonists induce memory-related disorders [16, 29, 36], whereas antagonists of these receptors facilitate memory and learning processes [16, 29, 37–39]. Moreover, CB1 receptor agonists might provoke psychosis-like symptoms, in turn, CB1 receptor antagonists show antipsychotic properties assessed in animal models of schizophrenia [40–42]. Similarly, there is evidence that the CB2 receptors are also involved in the psychosis-like effects [17, 43–45].

Naturally, the third component of the ECS system, i.e., endocannabinoids and enzymes responsible for the metabolism of endocannabinoids (FAAH and MAGL), is also important in the context of schizophrenia-like effects [15, 22]. Assuming that the role of the hydrolase inhibitors in the schizophrenia-like responses has not been fully elucidated yet, the purpose of the experiments was to explore the role of the ECS through inhibition of enzymes degrading endocannabinoids in the brain, in the various symptoms of schizophrenia. Among all modulators of enzyme-metabolizing endocannabinoids, in the present experiments, we used two compounds: URB 597 and JZL 184. The first one exhibits the characteristics of FAAH inhibitor, which is the main factor of AEA degradation [46], while the second compound acts by carbonylation of nucleophilic groups and leads to MAGL blockade, which can increase the concentration of 2-AG [47]. For the first time to our knowledge, we assessed the influence of both inhibitors on the positive and cognitive schizophrenia-like symptoms in mice. We determined the involvement of URB 597 and JZL 184 on the MK-801-induced hyperlocomotor activity or memory impairment in mice, which correlates with psychotic and cognitive symptoms of schizophrenia in humans, respectively. Assessment of cognitive processes was carried out using the PA test; positive symptoms of schizophrenia measured as hyperlocomotion were assessed in actimeters.

In the first step of our experiments, we revealed that both an acute administration of URB 597 (0.1 mg/kg) as well as JZL 184 (4 mg/kg) improved memory and learning processes in the PA test in mice. Moreover, an acute injection of both inhibitors, e.g., URB 597 (0.1 and 0.3 mg/kg) or JZL 184 (4–40 mg/kg) induced dose-dependently hypolocomotion in mice assessed in actimeters. The next set of our experiments indicated that an acute administration of URB 597 (0.3 mg/kg) attenuated MK-801 (0.6 mg/kg)-induced memory impairment. In turn, an acute administration of this inhibitor at a higher dose (1 mg/kg) potentiated MK-801 (0.3 mg/kg)-induced memory impairment. Similarly, the second tested inhibitor, JZL 184 (at two doses used, 20 and 40 mg/kg) intensified and potentiated this MK-801 (0.3 and 0.6 mg/kg)-provoked amnestic effects. Moreover, we revealed that an acute administration of URB 597 (1 mg/kg) had no statistically significant influence on the mice hyperactivity after MK-801 administration (0.3 as well as 0.6 mg/kg), whereas an acute injection of JZL 184 (1 mg/kg) before MK-801 (0.3 and 0.6 mg/kg) intensified MK-801-provoked hyperlocomotion in mice.

Recent available studies reported that FAAH or MAGL inhibitors increase endogenous levels of the CB receptor agonist, AEA and 2-AG, and might represent a promising tool for treating a wide range of disorders with minimal risk of adverse cannabis-like side effects. Some FAAH inhibitors, including URB 694, PF-04457845, and AM 3506 [48], have shown moderate to strong reinforcing effects. However, another FAAH inhibitor, URB 597, does not produce classical THC-like effects such as catalepsy, hyperthermia, and hyperphagia [49]. This compound also shows no signs of abuse potential in animal models of cannabis abuse [50], but in turn, has been shown to display activity in a rodent model of inflammatory, neuropathic pain as well as anxiety and depression, and has been found to enhance non-opioid stress-induced analgesia [51, 52].

It has been also demonstrated that direct (CB1 agonist) and indirect (FAAH and MAGL inhibitor) activation can impair cognitive performance of animals in a variety of memory assays [53, 54]. Controversially, some studies report memory-enhancing effects of inhibitors mentioned above. Ratano with co-workers [55, 56] showed that enhancing AEA levels facilitates memory consolidation for aversive events through a concurrent activation of both CB1 and CB2 receptors.

However, the influence on the memory by indirect activation of the CB1 receptor (via FAAH and MAGL inhibition) often depends on dose, experimental procedure, or other environmental factors. In the context of our experiments, an interest seems to be a data concerning the influence of FAAH or MAGL inhibition on the memory and learning processes. Ratano et al. [56], already cited, showed that JZL 184 enhanced memory consolidation. These effects are also in line with previous evidence showing that pharmacological or genetic inactivation of 2-AG metabolism improved memory performances in the variety of the animal model of memory, like the Morris water maze paradigm and novel object recognition test [57]. Moreover, it has been reported that both direct activation of CB receptors or increased AEA signaling, through inhibition of its metabolizing enzyme (FAAH), enhances consolidation and reconsolidation of aversive memories [58].

Although URB 597 as a FAAH inhibitor has been studied most intensively [59–61], its precise influence on memory or other schizophrenia-like effects has not been fully characterized. Some available reports concerning this subject seem also controversial. Inhibition or genetic deletion of FAAH, which substantially increases endogenous levels of AEA, has been found to enhance rather than impair memory in rodents trained with procedures involving aversively motivated behavior (i.e., water Morris maze test) [54, 62, 63], or passive avoidance with a context associated with foot shock [64–66], in accordance with our results. In turn, other memory-related studies have mostly shown impairment rather enhancement after treatment with a FAAH inhibitor (URB 597) [24, 67]. It appears that aversively motivated learning is most sensitive to be enhanced by FAAH manipulations, possibly due to the effects of FAAH inhibition on anxiety-related responses [68] or coping behavior [69]. Moreover, URB 597 (0.3 and 1 mg/kg) treatment could alleviate the negative influence of WIN 55,212-2, a partial CB1 receptor agonist, on cognition and memory. These results indicate a potential of URB 597 to protect against memory deficits induced by cannabinoids [64].

As we mentioned, the indirect modulating effect of URB 597 occurs through a concurrent activation of both CB1 and CB2 receptors, and is associated with an increase in AEA concentration due to the inhibition of FAAH-induced hydrolysis. AEA, mainly present in the central nervous system, has a significant influence on the control of physiological activities, including those concerning cognitive abilities [70]. It has been proved that in addition to the anxiolytic effect observed, URB 597, by regulating AEA concentration, has a significant impact on the consolidation of memory processes [24]. This is extremely important in the context of research on cognitive symptoms representative for schizophrenia. An additional confirmation of the association of the pharmacological activity of URB 597 with this disease has been provided by studies which show a beneficial effect of URB 597 administration to rats with negative symptoms induced by chronic PCP exposure [71]. However, the experiment described in the abovementioned paper shows that the therapeutic effect only occurs in animals with already induced symptoms. In case of animals not exposed to substances provoking symptoms correlating with schizophrenia, repeated administration of URB 597 causes the memory deficits which was related to ECS system disorder. Nevertheless, it proves a significant difference between ECS dysfunctions and the etiology of the disease [71].

In our experiments, we showed that a single injection of URB 597, at the ineffective dose of 0.3 mg/kg, significantly affected the MK-801 (0.6 mg/kg)-induced amnesic effect in the PA test in mice, amplifying this effect in comparison to the control group. This effect correlates with results showing that the administration of URB 597 results in the induction of memory acquisition disorders, as a result of indirect regulation of the concentration of AEA, which has a high affinity for CB receptors. As described in our previous articles [16, 17], CB1 receptor agonists induce symptoms typical for schizophrenia, positive and cognitive ones. Their influence on disorders related to cognition has been repeatedly described, both for natural and synthetic cannabinoids. In this case, it should be noted that AEA is the main CB1 receptor agonist, whose activation causes a decrease in cAMP, leading to the closure of Ca^2+^ channels. The following inhibitory effect on membrane-depolarization-induced activity leads to blockade of neurotransmitter release [72]. A research which proves that the administration of CB1 receptor antagonist, such as AM-251, reverses the MK-801-induced hyperlocomotion could be a possible confirmation of this theory [16].

Given all these data cited, it appears that the simultaneous administration of URB 597 and MK-801 is an example of synergism. CB1 receptor agonists, including AEA, whose concentration has been increased by the administration of URB 597, through the mechanism described earlier, blocks the release of Glu, which is the main neurotransmitter of the glutamatergic system. This may trigger an additional reduction in the NMDA activity, also blocked by its antagonist like MK-801. This type of dual mechanism of pharmacodynamics may cause that an administration of URB 597 may intensify the amnestic symptoms induced by MK-801.

Thus, based on our experiments, we can also speculate that the contribution of the FAAH-endocannabinoid system may depend on the level of stress associated with environmental conditions. Observed influence of URB 597 indicates that FAAH inhibitors can vary considerably in their effect profiles and should be evaluated individually for specific therapeutic and adverse effects. In the future, our results may also lead to a better understanding of the brain endocannabinoids-related mechanisms underlying schizophrenia.

The usefulness of FAAH inhibitors remains uncertain; thus, recent studies have also proposed that MAGL inhibitors may be novel modulators for symptoms of schizophrenia. The currently most potent and the most selective of the known MAGL inhibitors, JZL 184, was created as a modification of the previously known molecule called JZL 175. Due to changes in the chemical structure, it shows the ability of almost complete blockade of the enzyme responsible for the degradation of 2-AG, which is an endogenous agonist of the CB1 receptors. This results in an eightfold increase in 2-AG concentration without affecting the AEA level. Not without significance is the fact that there is no affinity for the CB1 receptor, which is an additional advantage when conducting experiments aimed at testing endocannabinoids [47]. What is of interest, JZL 184 has been shown to produce a long-lasting elevation of 2-AG, as well as cannabinoid-like behavioral responses in mice or in rats [47, 73, 74].

It should be pointed out that an administration of the MAGL inhibitor, JZL 184, dose-dependently decreases somatic and aversive sings of nicotine withdrawal. Furthermore, these protective effects of JZL 184 were blocked by rimonabant, a CB1 receptor antagonist, suggesting a CB1-mediated mechanism [75]. Concerning the cognitive processes, JZL 184 did not affect memory in an object recognition procedure [24]; thus, both JZL 184 and a dual FAAH-MAGL inhibitor, JZL 185, have impaired memory in a repeated acquisition water maze procedure in mice [54]. It has been also found that JZL 184 (0.5–1 mg/kg) reduces traumatic memory recall in an animal model of post-traumatic stress disorder when administered 1 h before extinction sessions [66]. Our findings demonstrated that pharmacological inhibition of MAGL through JZL 184 increased latency index in the PA test at testing at the dose 4 mg/kg, whereas the higher doses (8–40 mg) did not exert any effect. Similarly, we have observed the memory improvement after injection of URB 597 at the dose of 0.1 mg/kg but not after the higher doses (0.3 and 1 mg/kg). On the other hand, other studies have demonstrated that enhanced levels of 2-AG impair spatial memory retrieval [66] and impaired extinction of fear memory [76]. These controversial effects are very common in memory research especially for cannabinoid compounds, as already stated.

Our experiments also revealed that JZL 184 induced hypolocomotion, dose-dependently. In agreement with these studies, it has been found, in rats and in mice, that JZL 184 produces a significant inhibition of motor activity at the dose of 30 mg/kg, and a more importantly, it elevates 2-AG at the highest doses (15 and 30 mg/kg) [47]. On the other hand, injection of JZL 184 at its highest dose (30 mg/kg) produces a significant suppression of locomotor activity that was not reversed by either SR 141716A or AM 251, CB receptor antagonists, even though both compounds were given at doses known to produce behavioral effects in rats [73]. In addition, there are no correlations between locomotor activity and 2-AG levels in all brain areas examined. These observations confirm the previous study carried out by Long et al. in mice showing that JZL 184 induces 2-AG elevation and hypomotility [47].

The pharmacological characteristics of JZL 184 result from the indirect modulation of CB1 receptor activity [77]. This action has a significant effect on the number of neurotransmitters released, including those directly related to the alleged etiology of schizophrenia. Particularly, noteworthy are articles investigating the effect of JZL 184 on the hippocampus functions. A significant correlation was observed between 2-AG activity and synaptic dysfunctions leading to cognitive impairment, including processes related to memory acquisition caused by the modulation of releasing the relays in the GABA-ergic and glutamatergic systems, triggered by the activation of CB1 receptors, specifically by 2-AG. As we previously mentioned, receptor stimulation leads to the inhibition of a neurotransmission release, which results from blocking Ca^2+^ channels while opening K^+^ channels [78].

Accordingly, it’s commonly accepted that JZL 184 via blocking MAGL activity, contributes to the inhibition of the metabolism of 2-AG. Thus, the concentration of 2-AG in the central nervous system is significantly higher than of AEA. 2-AG, being one of the main factors that induce presynaptic inhibition of neurotransmitter release due to membrane depolarization, is also largely associated with the functionality of other structures, as is the case of AEA. However, the difference resulting from the nature of both ligands (AEA, as a partial agonist and 2-AG as a full agonist of CB1 receptors) seems not to be without significance [79, 80].

Particularly, noteworthy is the literature data connecting the level of 2-AG with the inhibition of the activity of the GABA-ergic system, which is the main inhibitory pathway in the central nervous system. Although drugs that increase GABA activity induce amnesia, in contrast to results obtained in the experiments, this structure is directly related to the NMDA receptor activity, considered to be the main central nervous system activating receptor [81]. The symptoms are induced only after the combined administration of ligands, along with MK-801. This proves that the endocannabinoid itself, including 2-AG, is not able to induce symptoms associated with schizophrenia, but only to intensify them by affecting the dysfunctional glutamatergic system. The confirmation of this theory is a result of our previous experiments using the CB2 receptor ligands (e.g., JWH 133 and AM 630) which potentiated the MK-801-induced hyperactivity [17].

Our results allow evaluating of the possible relationship between ECS and positive or cognitive symptoms of schizophrenia, focusing on the indirect modulation of ECS functioning, using FAAH or MAGL inhibitors. Based on the results from our experiments, we can confirm that the ECS may be a key element involved in the development of schizophrenia. In clinical studies, this assumption was supported by several observations showing that cannabis use is associated with an increased risk of developing schizophrenia or that schizophrenia-like symptoms may develop in non-schizophrenic cannabis users [82, 83]. In addition, schizophrenic patients showed both positive and negative symptoms and cognitive deficits after administration of delta-9-THC [15, 84].

## Conclusion

Our research confirms that there is a close correlation between the activity of the ECS and the occurrence of schizophrenia symptoms. Administration of inhibitors in the presence of schizophrenia-related factors, e.g., dysfunction of the activity of the GABA system intensifies the cognitive and positive symptoms of schizophrenia in mice. These findings might have a high diagnostic aspect in the future studies.

On the other hand, it can be speculated that the research concerning FAAH or MAGL inhibition may open a new approach for developing medications that act indirectly by enhancing the actions of endogenous lipid amide mediators. It is worth mentioning that FAAH and MAGL inhibition might be related to a wide spectrum of therapeutic actions in cognitive-related disorders. However, further studies are necessary to identify the clear mechanisms underlying the action of selective inhibitors that can be used as pharmacological tools to manipulate AEA and 2-AG signaling independently and to study their possible interactions.

## References

[1] Kohen D (2004). Diabetes mellitus and schizophrenia: historical perspective. Br J Psychiatry.

[2] Cirillo MA, Seidman LJ (2003). Verbal declarative memory dysfunction in schizophrenia: from clinical assessment to genetics and brain mechanisms. Neuropsychol Rev.

[3] Goldman-Rakic PS (1994). Working memory dysfunction in schizophrenia. J Neuropsychiatry Clin Neurosci.

[4] Krystal JH, Anand A, Moghaddam B (2002). Effects of NMDA receptor antagonists: implications for the pathophysiology of schizophrenia. Arch Gen Psychiatry.

[5] Moghaddam B, Javitt D (2012). From revolution to evolution: the glutamate hypothesis of schizophrenia and its implication for treatment. Neuropsychopharmacology.

[6] Benes FM, Vincent SL, Marie A, Khan Y (1996). Up-regulation of GABA A receptor binding on neurons of the prefrontal cortex in schizophrenic subjects. Neuroscience.

[7] Javitt DC, Steinschneider M, Schroeder CE, Arezzo JC (1996). Role of cortical N-methyl-D-aspartate receptors in auditory sensory memory and mismatch negativity generation: implications for schizophrenia. Proc Natl Acad Sci U S A.

[8] Harrison PJ, Law AJ, Eastwood SL (2003). Glutamate receptors and transporters in the hippocampus in schizophrenia. Ann N Y Acad Sci.

[9] Glausier JR, Lewis DA (2013). Dendritic spine pathology in schizophrenia. Neuroscience.

[10] Geyer M, Moghaddam B, Davis KL, Charney C, Coyle JT, Nemeroff C (2002). Animal models relevant to schizophrenia disorder. Psychopharmacology: the Fifth Generation of Progress.

[11] Kilts CD (2001). The changing roles and targets for animal models of schizophrenia. Biol Psychiatry.

[12] Di Marzo V, De Petrocellis L (2010). Endocannabinoids as regulators of transient receptor potential (TRP) channels: a further opportunity to develop new endocannabinoid-based therapeutic drugs. Curr Med Chem.

[13] O'Sullivan SE (2007). Cannabinoids go nuclear: evidence for activation of peroxisome proliferator-activated receptors. Br J Pharmacol.

[14] Dinh TP, Carpenter D, Leslie FM, Freund TF, Katona I, Sensi SL, Kathuria S, Piomelli D (2002). Brain monoglyceride lipase participating in endocannabinoid inactivation. Proc Natl Acad Sci U S A.

[15] Leweke FM, Mueller JK, Lange B, Rohleder C (2016). Therapeutic potential of cannabinoids in psychosis. Biol Psychiatry.

[16] Kruk-Slomka M, Budzynska B, Slomka T, Banaszkiewicz I, Biala G (2016). The influence of the CB1 receptor ligands on the schizophrenia-like effects in mice induced by MK-801. Neurotox Res.

[17] Kruk-Slomka M, Banaszkiewicz I, Biala G (2017). The impact of CB2 receptor ligands on the MK-801-induced hyperactivity in mice. Neurotox Res.

[18] Clapper JR, Mangieri RA, Piomelli D (2009). The endocannabinoid system as a target for the treatment of cannabis dependence. Neuropharmacology.

[19] Hlavacova N, Chmelova M, Danevova V, Csanova A, Jezova D (2015). Inhibition of fatty-acid amide hydrolyse (FAAH) exerts cognitive improvements in male but not female rats. Endocr Regul.

[20] Luque-Rojas MJ, Galeano P, Suárez J, Araos P, Santín LJ, de Fonseca FR, Calvo EB (2013). Hyperactivity induced by the dopamine D2/D3 receptor agonist quinpirole is attenuated by inhibitors of endocannabinoid degradation in mice. Int J Neuropsychopharmacol.

[21] Panlilio LV, Thorndike EB, Nikas SP, Alapafuja SO, Bandiera T, Cravatt BF, Makriyannis A, Piomelli D, Goldberg SR, Justinova Z (2016). Effects of fatty acid amide hydrolase (FAAH) inhibitors on working memory in rats. Psychopharmacology.

[22] Seillier A, Advani T, Cassano T, Hensler JG, Giuffrida A (2010). Inhibition of fatty-acid amide hydrolase and CB1 receptor antagonism differentially affect behavioural responses in normal and PCP-treated rats. Int J Neuropsychopharmacol.

[23] Bubenikova-Valesova V, Svoboda J, Horacek J, Sumiyoshi T (2010). Effect of tandospirone, a serotonin-1A receptor partial agonist, on information processing and locomotion in dizocilpine-treated rats. Psychopharmacology.

[24] Busquets-Garcia A, Puighermanal E, Pastor A, de la Torre R, Maldonado R, Ozaita A (2011). Differential role of anandamide and 2-arachidonoylglycerol in memory and anxiety-like responses. Biol Psychiatry.

[25] Javadi-Paydar M, Zakeri A, Norouzi H, Rastegar N, Mirazi A (2012). Involvement of nitric oxide in granisetron improving effect on scopolamine-induced memory impairment in mice. Brain Res Rev.

[26] Lysenko LV, Kim J, Henry C, Tyrtyshnaia A, Kohnz RA, Madamba F, Simon GM, Kleschevnikova NE, Nomura DK, Ezekowitz RA, Kleschevnikov AM (2014). Monoacylglycerol lipase inhibitor JZL184 improves behavior and neural properties in Ts65Dn mice, a model of down syndrome. PLoS One.

[27] Nestler EJ, Hyman SE (2010). Animal models of neuropsychiatric disorders. Nat Neurosci.

[28] Zhou Y, Liu MD, Fan Y, Ding JH, Du RH, Hu G (2012). Enhanced MK-801-induced locomotion in Kir6.2 knockout mice. Neurosci Res.

[29] Kruk-Slomka M, Biala G (2016). CB1 receptors in the formation of the different phases of memory-related processes in the inhibitory avoidance test in mice. Behav Brain Res.

[30] Mohn AR, Gainetdinov RR, Caron MG, Koller BH (1999). Mice with reduced NMDA receptor expression display behaviors related to schizophrenia. Cell.

[31] Chimakurthy J, Talasila M (2010). Effects of curcumin on pentylenetetrazole-induced anxiety-like behaviors and associated changes in cognition and monoamine levels. Psychol Neurosci.

[32] Castillo PE, Younts TJ, Chávez AE, Hashimotodani Y (2012). Endocannabinoid signaling and synaptic function. Neuron.

[33] Demuth DG, Molleman A (2006). Cannabinoid signaling. Lif Sci.

[34] Howlett AC, Breivogel CS, Childers SR, Deadwyler SA, Hampson RE, Porrino LJ (2004). Cannabinoid physiology and pharmacology: 30 years of progress. Neuropharmacology.

[35] Kucerova J, Tabiova K, Drago F, Micale V (2014). Therapeutic potential of cannabinoids in schizophrenia. Recent Pat CNS Drug Discov.

[36] Ferrari F, Ottani A, Vivoli R, Giuliani D (1999). Learning impairment produced in rats by the cannabinoid agonist HU 210 in a water-maze task. Pharmacol Biochem Behav.

[37] Lichtman AH, Peart J, Poklis JL, Bridgen DT, Razdan RK, Wilson DM, Poklis A, Meng Y, Byron PR, Martin BR (2000). Pharmacological evaluation of aerosolized cannabinoids in mice. Eur J Pharmacol.

[38] Takahashi RN, Pamplona FA, Fernandes MS (2005). The cannabinoid antagonist SR 141716A facilitates memory acquisition and consolidation in the mouse elevated T-maze. Neurosci Lett.

[39] Terranova JP, Storme JJ, Lafon N, Peño A, Rinaldi-Carmona M (1996). Improvement of memory in rodents by the selective CB1 cannabinoid antagonist, SR 141716. Psychopharmacology.

[40] Almeida V, Peres FF, Levin R, Suiama MA, Calzavara MB, Zuardi AW, Hallak JE, Crippa JA, Abílio VC (2014). Effects of cannabinoid and vanilloid drugs on positive and negative-like symptoms on an animal model of schizophrenia: the SHR strain. Schizophr Res.

[41] Levin R, Almeida V, Peres FF, Calzavara MB, da Silva ND, Suiama MA, Niigaki ST, Zuardi AW, Hallak JE, Crippa JA, Abílio VC (2012). Antipsychotic profile of cannabidiol and rimonabant in an animal model of emotional context processing in schizophrenia. Curr Pharm Des.

[42] Roser P, Haussleiter IS (2012). Antipsychotic-like effects of cannabidiol and rimonabant: systematic review of animal and human studies. Curr Pharm Des.

[43] Ishiguro H, Horiuchi Y, Ishikawa M, Koga M, Imai K, Suzuki Y, Morikawa M, Inada T, Watanabe Y, Takahashi M, Someya T, Ujike H, Iwata N, Ozaki N, Onaivi ES, Kunugi H, Sasaki T, Itokawa M, Arai M, Niizato K, Iritani S, Naka I, Ohashi J, Kakita A, Takahashi H, Nawa H, Arinami T (2010). Brain cannabinoid CB2 receptor in schizophrenia. Biol Psychiatry.

[44] Khella R, Short JL, Malone DT (2014). CB2 receptor agonism reverses MK-801-induced disruptions of prepulse inhibition in mice. Psychopharmacology.

[45] Ortega-Alvaro A, Aracil-Fernández A, García-Gutiérrez MS, Navarrete F, Manzanares J (2011). Deletion of CB2 cannabinoid receptor induces schizophrenia-related behaviors in mice. Neuropsychopharmacology.

[46] Butini S, Brindisi M, Gemma S, Minetti P, Cabri W, Gallo G, Vincenti S, Talamonti E, Borsini F, Caprioli A, Stasi MA, Di Serio S, Ros S, Borrelli G, Maramai S, Fezza F, Campiani G, Maccarrone M (2012). Discovery of potent inhibitors of human and mouse fatty acid amide hydrolases. J Med Chem.

[47] Long JZ, Li W, Booker L, Burston JJ, Kinsey SG, Schlosburg JE, Pavón FJ, Serrano AM, Selley DE, Parsons LH, Lichtman AH, Cravatt BF (2009). Selective blockade of 2-arachidonoylglycerol hydrolysis produces cannabinoid behavioral effects. Nat Chem Biol.

[48] Justinova Z, Panlilio LV, Moreno-Sanz G, Redhi GH, Auber A, Secci ME, Mascia P, Bandiera T, Armirotti A, Bertorelli R, Chefer SI, Barnes C, Yasar S, Piomelli D, Goldberg SR (2015). Effects of fatty acid amide hydrolase (FAAH) inhibitors in non-human primate models of nicotine reward and relapse. Neuropsychopharmacology.

[49] Kathuria S, Gaetani S, Fegley D, Valiño F, Duranti A, Tontini A, Mor M, Tarzia G, La Rana G, Calignano A, Giustino A, Tattoli M, Palmery M, Cuomo V, Piomelli D (2003). Modulation of anxiety through blockade of anandamide hydrolysis. Nat Med.

[50] Gobbi G, Bambico FR, Mangieri R, Bortolato M, Campolongo P, Solinas M, Cassano T, Morgese MG, Debonnel G, Duranti A, Tontini A, Tarzia G, Mor M, Trezza V, Goldberg SR, Cuomo V, Piomelli D (2005). Antidepressant-like activity and modulation of brain monoaminergic transmission by blockade of anandamide hydrolysis. Proc Natl Acad Sci U S A.

[51] Bambico FR, Duranti A, Tontini A, Tarzia G, Gobbi G (2009). Endocannabinoids in the treatment of mood disorders: evidence from animal models. Curr Pharm Des.

[52] McLaughlin RJ, Gobbi G (2012). Cannabinoids and emotionality: a neuroanatomical perspective. Neuroscience.

[53] Griebel G, Pichat P, Beeské S, Leroy T, Redon N, Jacquet A, Françon D, Bert L, Even L, Lopez-Grancha M, Tolstykh T, Sun F, Yu Q, Brittain S, Arlt H, He T, Zhang B, Wiederschain D, Bertrand T, Houtmann J, Rak A, Vallée F, Michot N, Augé F, Menet V, Bergis OE, George P, Avenet P, Mikol V, Didier M, Escoubet J (2015). Selective blockade of the hydrolysis of the endocannabinoid 2-arachidonoylglycerol impairs learning and memory performance while producing antinociceptive activity in rodents. Sci Rep.

[54] Wise LE, Long KA, Abdullah RA, Long JZ, Cravatt BF, Lichtman AH (2012). Dual fatty acid amide hydrolase and monoacylglycerol lipase blockade produces THC-like Morris water maze deficits in mice. ACS Chem Neurosci.

[55] Ratano P, Palmery M, Trezza V, Campolongo P (2017). Cannabinoid modulation of memory consolidation in rats: beyond the role of cannabinoid receptor subtype 1. Front Pharmacol.

[56] Ratano P, Petrella C, Forti F, Passeri PP, Morena M, Palmery M, Trezza V, Severini C, Campolongo P (2018). Pharmacological inhibition of 2-arachidonoilglycerol hydrolysis enhances memory consolidation in rats through CB2 receptor activation and mTOR signaling modulation. Neuropharmacology.

[57] Pan B, Wang W, Zhong P, Blankman JL, Cravatt BF, Liu QS (2011). Alterations of endocannabinoid signaling, synaptic plasticity, learning, and memory in monoacylglycerol lipase knock-out mice. J Neurosci.

[58] Morena M, Campolongo P (2014). The endocannabinoid system: an emotional buffer in the modulation of memory function. Neurobiol Learn Mem.

[59] Jayamanne A, Greenwood R, Mitchell VA, Aslan S, Piomelli D, Vaughan CW (2006). Actions of the FAAH inhibitor URB597 in neuropathic and inflammatory chronic pain models. Br J Pharmacol.

[60] Justinova Z, Mangieri RA, Bortolato M, Chefer SI, Mukhin AG, Clapper JR, King AR, Redhi GH, Yasar S, Piomelli D, Goldberg SR (2008). Fatty acid amide hydrolase inhibition heightens anandamide signaling without producing reinforcing effects in primates. Biol Psychiatry.

[61] Scherma M, Panlilio LV, Fadda P, Fattore L, Gamaleddin I, Le Foll B, Justinová Z, Mikics E, Haller J, Medalie J, Stroik J, Barnes C, Yasar S, Tanda G, Piomelli D, Fratta W, Goldberg SR (2008). Inhibition of anandamide hydrolysis by URB597 reverses abuse-related behavioral and neurochemical effects of nicotine in rats. J Pharmacol Exp Ther.

[62] Varvel SA, Wise LE, Niyuhire F, Cravatt BF, Lichtman AH (2007). Inhibition of fatty-acid amide hydrolase accelerates acquisition and extinction rates in a spatial memory task. Neuropsychopharmacology.

[63] Wise LE, Harloe JP, Lichtman AH (2009). Fatty acid amide hydrolase (FAAH) knockout mice exhibit enhanced acquisition of an aversive, but not of an appetitive, Barnes maze task. Neurobiol Learn Mem.

[64] Hasanein P, Teimuri Far M (2015). Effects of URB597 as an inhibitor of fatty acid amide hydrolase on WIN55, 212-2-induced learning and memory deficits in rats. Pharmacol Biochem Behav.

[65] Mazzola C, Medalie J, Scherma M, Panlilio LV, Solinas M, Tanda G, Drago F, Cadet JL, Goldberg SR, Yasar S (2009). Fatty acid amide hydrolase (FAAH) inhibition enhances memory acquisition through activation of PPAR-alpha nuclear receptors. Learn Mem.

[66] Morena M, Roozendaal B, Trezza V, Ratano P, Peloso A, Hauer D, Atsak P, Trabace L, Cuomo V, McGaugh JL, Schelling G, Campolongo P (2014). Endogenous cannabinoid release within prefrontal-limbic pathways affects memory consolidation of emotional training. Proc Natl Acad Sci U S A.

[67] Basavarajappa BS, Nagre NN, Xie S, Subbanna S (2014). Elevation of endogenous anandamide impairs LTP, learning, and memory through CB1 receptor signaling in mice. Hippocampus.

[68] Haller J, Barna I, Barsvari B, Gyimesi Pelczer K, Yasar S, Panlilio LV, Goldberg S (2009). Interactions between environmental aversiveness and the anxiolytic effects of enhanced cannabinoid signaling by FAAH inhibition in rats. Psychopharmacology.

[69] Haller J, Goldberg SR, Pelczer KG, Aliczki M, Panlilio LV (2013). The effects of anandamide signaling enhanced by the FAAH inhibitor URB597 on coping styles in rats. Psychopharmacology.

[70] Mallet P. E., Beninger R. J. (1996). The endogenous cannabinoid receptor agonist anandamide impairs memory in rats. Behavioural Pharmacology.

[71] Seillier A, Martinez AA, Giuffrida A (2013). Phencyclidine-induced social withdrawal results from deficient stimulation of cannabinoid CB_1_ receptors: implications for schizophrenia. Neuropsychopharmacology.

[72] Hampson RE, Miller F, Palchik G, Deadwyler SA (2011). Cannabinoid receptor activation modifies NMDA receptor mediated release of intracellular calcium: implications for endocannabinoid control of hippocampal neural plasticity. Neuropharmacology.

[73] Seillier A, Dominguez Aguilar D, Giuffrida A (2014). The dual FAAH/MAGL inhibitor JZL195 has enhanced effects on endocannabinoid transmission and motor behavior in rats as compared to those of the MAGL inhibitor JZL184. Pharmacol Biochem Behav.

[74] Steinmetz AB, Freeman JH (2016). Cannabinoid modulation of memory consolidation within the cerebellum. Neurobiol Learn Mem.

[75] Muldoon PP, Chen J, Harenza JL, Abdullah RA, Sim-Selley LJ, Cravatt BF, Miles MF, Chen X, Lichtman AH, Damaj MI (2015). Inhibition of monoacylglycerol lipase reduces nicotine withdrawal. Br J Pharmacol.

[76] Hartley ND, Gunduz-Cinar O, Halladay L, Bukalo O, Holmes A, Patel S (2016). 2-arachidonoylglycerol signaling impairs short-term fear extinction. Transl Psychiatry.

[77] Labar G, Wouters J, Lambert DM (2010). A review on the monoacylglycerol lipase: at the interface between fat and endocannabinoid signalling. Curr Med Chem.

[78] Du H, Kwon IK, Kim J (2013). Neuregulin-1 impairs the long-term depression of hippocampal inhibitory synapses by facilitating the degradation of endocannabinoid 2-AG. J Neurosci.

[79] Mechoulam R, Ben-Shabat S, Hanus L, Ligumsky M, Kaminski NE, Schatz AR, Gopher A, Almog S, Martin BR, Compton DR (1995). Identification of an endogenous 2-monoglyceride, present in canine gut, that binds to cannabinoid receptors. Biochem Pharmacol.

[80] Sugiura T, Kodaka T, Nakane S, Miyashita T, Kondo S, Suhara Y, Takayama H, Waku K, Seki C, Baba N, Ishima Y (1999). Evidence that the cannabinoid CB1 receptor is a 2-arachidonoylglycerol receptor. Structure-activity relationship of 2-arachidonoylglycerol, ether-linked analogues, and related compounds. J Biol Chem.

[81] Volk DW, Eggan SM, Lewis DA (2010). Alterations in metabotropic glutamate receptor 1α and regulator of G protein signaling 4 in the prefrontal cortex in schizophrenia. Am J Psychiatry.

[82] Gage SH, Hickman M, Zammit S (2016). Association between cannabis and psychosis: epidemiologic evidence. Biol Psychiatry.

[83] Shrivastava A, Johnston M, Terpstra K, Bureau Y (2015). Pathways to psychosis in cannabis abuse. Clin Schizophr Relat Psychoses.

[84] D’Souza DC, Abi-Saab WM, Madonick S, Forselius-Bielen K, Doersch A, Braley G, Gueorguieva R, Cooper TB, Krystal JH (2005). Delta-9-tetrahydrocannabinol effects in schizophrenia: implications for cognition, psychosis, and addiction. Biol Psychiatry.

